# Evernic Acid: A Low‐Toxic and Selective Alternative to Chemotherapeutic Agents in the Treatment of Ovarian Cancer

**DOI:** 10.1002/ardp.70015

**Published:** 2025-05-22

**Authors:** Mine Ensoy, Damla Nur Parıltı, Ayşe Hale Alkan, Kübra Nur Kaplan İlhan, Pelin Mutlu, Bala Gür Dedeoğlu, Demet Cansaran‐Duman

**Affiliations:** ^1^ Biotechnology Institute Ankara University Keçiören Ankara Türkiye

**Keywords:** anticancer, apoptosis, evernic acid, ovarian cancer

## Abstract

Evernic acid (EA) has emerged as a potential therapeutic agent with its low toxicity and anticancer properties. In this study, the anticancer effect of EA on ovarian cancer cell lines and normal ovarian surface epithelial cells (OSE) was evaluated. The antiproliferative effect of EA was evaluated by xCELLigence Real‐Time Cell analysis, colony formation assay, and acridine orange and DAPI staining methods. Genotoxicity analysis was performed by comet assay. The effect of EA on cell migration was analyzed by wound healing assay. The potential of EA to induce apoptosis was also determined by evaluating the changes in gene and protein expression levels by qRT‐PCR and Western blot analysis, respectively. EA was found to be a promising potential therapeutic agent for ovarian cancer without showing significant cytotoxic effect on normal cells. Furthermore, EA decreased the ability of ovarian cancer cells for migration, increased the rate of apoptosis by inhibiting BIRC5 and activating CASP3, triggered cell cycle arrest in the G2/M phase, and caused a decrease in mitochondrial membrane potential and genotoxic effects. The results have shown that EA could be an effective candidate molecule for ovarian cancer treatment.

## Graphical abstract

The potential therapeutic effects of evernic acid (EA) were investigated using functional in vitro assays and the evaluation of gene and protein expression levels on ovarian cancer cell lines. EA demonstrated antiproliferative effects on ovarian cancer cells, primarily through the activation of apoptosis at both cellular and molecular levels. The findings suggest that EA may serve as a promising candidate for ovarian cancer therapy.

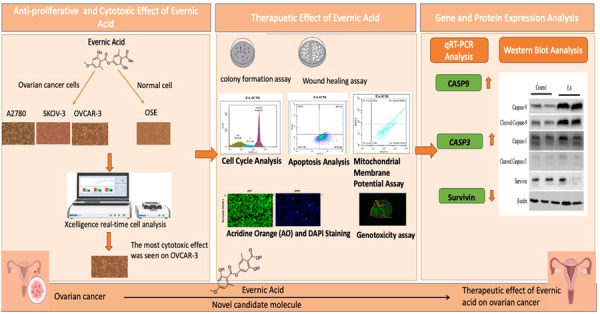

## 1 Introduction

Ovarian cancer (OC) is one of the most common and lethal cancer of the female reproductive system worldwide, which ranks 8th in mortality among the 10 most common cancers in women [1, 2]. The risk factors that were documented for ovarian cancer include a combination of genetic, lifestyle, and environmental factors, such as genetic factors and family history (mutations of BRCA1 and BRCA2 genes), fertility medication, smoking, obesity, hormonal therapy, and over‐ovulation [1, 3]. Ovarian cancer has in three primary forms: sex cord‐stromal, germ cell, and epithelial types of cancer [4]. About 90% of ovarian tumors are epithelial ovarian cancers (EOCs), making them the most prevalent type. There are four primary histotypes of EOC: serous, mucinous, clear cell, and endometrioid. High‐grade serous carcinoma (HGSC) and low‐grade serous carcinoma (LGSC) are two distinct histotypes of serous malignancies [5]. High‐grade serous ovarian cancer (HGSOC) has the highest mortality among all histotypes and is characterized by *TP53* and *BRCA1/2* mutations and high copy number alterations (CNAs) [6, 7]. OVCAR‐3 is a highly aggressive cell line and is generally used In Vitro and In Vivo drug resistance studies as an HGSOC model [6, 8]. OVCAR‐3 is resistant to chemotherapeutics commonly used for cancer treatment, such as paclitaxel [9], doxorubicin [7], cisplatin, melphalan, and adriamycin [10], and because of its tendency to develop resistance, this cell line is a highly suitable research model for ovarian cancer studies.

Chemotherapy, surgery, and radiotherapy are the commonly used treatments for advanced‐stage ovarian cancer patients [3]. Platinum‐based agents (carboplatin, cisplatin) and taxanes (paclitaxel, docetaxel) represent the most commonly used FDA‐approved chemotherapeutic agents in the treatment of ovarian cancer [11]. Several factors, such as the stage of the disease, the presence of biomarkers, and the patient's overall health, play a crucial role in determining the appropriate treatment approach [12].

Recent research emphasizes small drug candidates with fewer side effects and lower drug resistance [13, 14, 15, 16]. Small‐molecule drugs are advantageous due to their low molecular weight, predictable pharmacological properties, and cost‐effective production [17, 18, 19]. They enable targeted therapies with high bioavailability and selectivity. Since the FDA's approval of imatinib in 2001, 115 targeted small‐molecule cancer drugs have been clinically approved by 2023 [20, 21]. Poly(ADP‐ribose) polymerase (PARP) inhibitors are the most widely used FDA‐approved small molecule drugs for ovarian cancer. PARP inhibitors, which are associated with *BRCA1/2* mutations that result in homologous recombination DNA repair (HRR) deficiencies, are one of the treatment strategies used in targeted ovarian cancer therapy [22]. With the inhibition of PARP, single‐strand breaks (SSB) accumulate, which leads to double‐strand breaks (DSB) and eventually results in genomic instability and cell death for BRCA‐mutated cancer cells. Olaparib, niriparib, talazoparib, rucaparib, and veliparib are the commonly used PARP inhibitors in ovarian cancer treatment [22]. Despite the encouraging potential of PARP inhibitors as a cancer therapy, there are some inherent limitations, including resistance, toxicity, and their limited efficacy as therapeutic agents [23, 24]. In this regard, the present study aims to explore the necessity for the development of alternative treatment strategies, given the inadequacy of current treatment methods and their high toxicity profiles [25, 26].

The molecules under investigation in this study are derived from biological organisms and have the potential to provide innovative treatment options for challenging diseases such as ovarian cancer. These molecules may complement or replace existing treatment options based on their unique chemical structure, capacity to overcome resistance mechanisms, reduced toxicity, and effects on multiple biological targets. Evernic acid (EA), as a small molecule isolated from lichen species such as *Evernia prunastri* and *Pseudoevernia furfuraceae*, attracts attention with its antioxidant, antimicrobial, and anticancer activities [27, 28, 29, 30, 31, 32, 33, 34, 35]. EA is a metabolite of the depside class with two monoaromatic rings and has the chemical formula *C17H16O7*. EA has functional groups, such as hydroxyl, ester, and methyl groups, on two phenolic rings, and these groups contribute to hydrophobic interactions, polarity, and stability. There are various studies conducted on the effects of EA on different types of cancer, such as colon cancer and breast cancer [36, 37]. As a result of these studies, EA's low toxicity and specific effects on cancer cell lines, such as A2780, HeLa, MDA‐MB‐231, and MCF‐7, make it a potential therapeutic agent [34]. Nonetheless, the antiproliferative and apoptotic effects of EA on ovarian cancer have not yet been fully elucidated; therefore, it is important to investigate the therapeutic effects of EA small molecules.

The regulation of cell death is a critical area of interest in cellular biology due to its vital role in disease pathogenesis and its potential for therapeutic targeting. Cancer cells exploit various mechanisms to evade growth‐suppressive signals and evade apoptotic pathways, enabling their unregulated proliferation and contributing significantly to tumorigenesis [38]. Consequently, therapeutic strategies aimed at modulating apoptotic pathways represent a promising approach for the development of effective cancer treatments [39, 40].

In this study, the antiproliferative potential of EA small molecule was evaluated in relation to ovarian cancer cells (A2780, SKOV‐3, and OVCAR‐3) with different molecular subtypes and healthy ovarian surface epithelial cells (OSE). The therapeutic potential of the EA molecule was then investigated at the cellular, gene, and protein expression levels. The findings of this study indicate that the utilization of EA as a potential pharmaceutical agent in the treatment of ovarian cancer represents a significant advancement in overcoming the limitations of current therapeutic methodologies.

## 2 Results and Discussion

### 2.1 xCELLigence Real‐Time Cell Analysis

**Figure 1:**
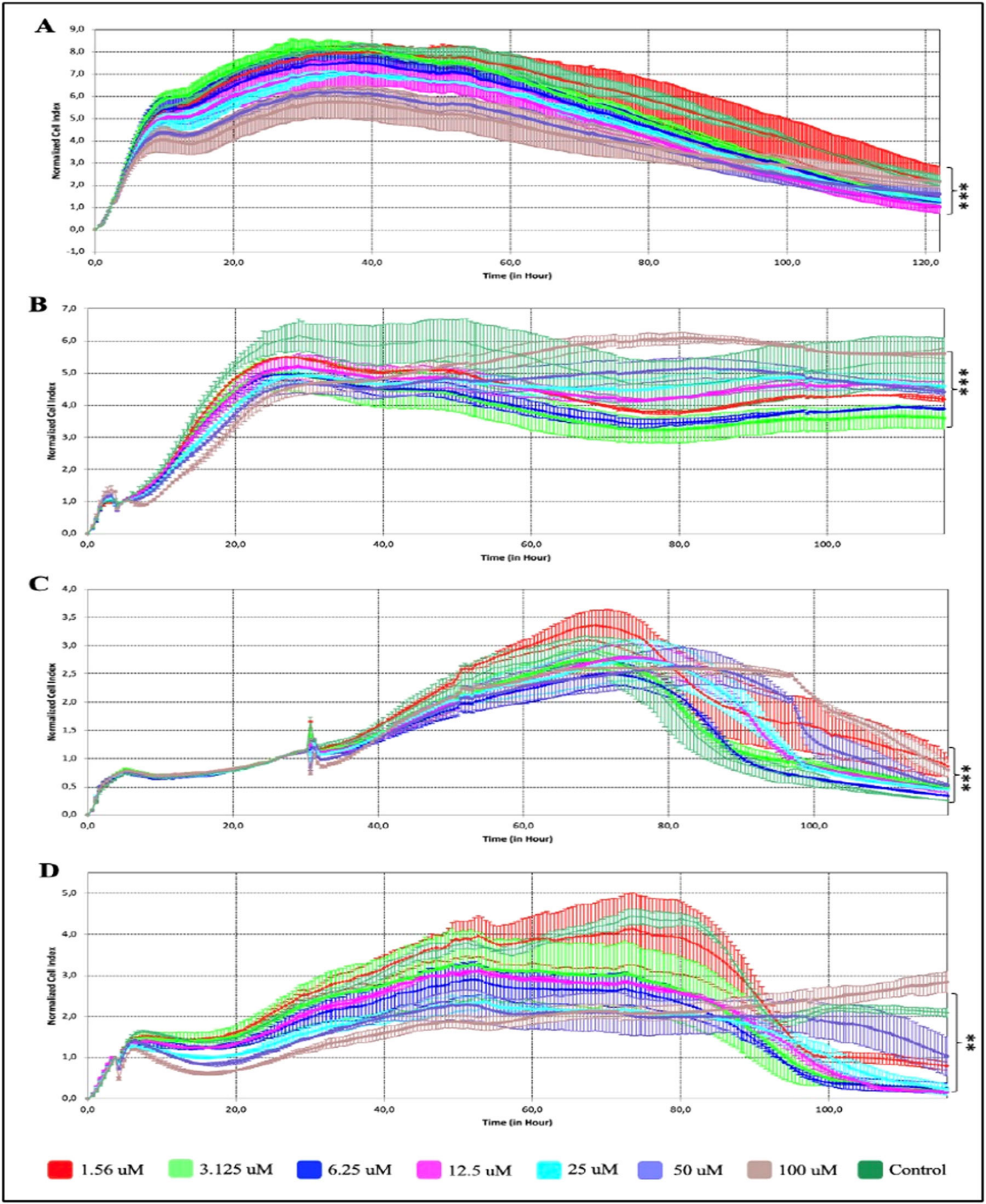
Dose‐ and time‐dependent antiproliferative effects of EA on (A) OVCAR‐3, (B) SKOV‐3, (C) A2780, and (D) OSE cells using the xCELLigence real‐time cell analyzer. (*t*‐test, **p < 0.05; ***p < 0.001).

Cellular proliferation was assessed using xCELLigence real‐time cell analysis to evaluate the antiproliferative effects of EA and determine the relative half‐maximal inhibitory concentrations (IC_50_) in A2780, SKOV‐3, OVCAR‐3, and OSE cells. Before EA treatment, cells were allowed to settle and grow for 24 h. At the end of 24 h, OVCAR‐3, SKOV‐3, A2780, and OSE cells were exposed to seven different concentrations of EA in the range of 1.56–100 µM and followed up for 120 h. The cell proliferation graphs and IC_50_ values were shown in Figure 1A–D and Table 1, respectively. When the antiproliferative effect of EA on ovarian cancer cells was examined, the IC_50_ value was determined to be 10 µM at 60 h in OVCAR‐3, 124 µM at 68 h in SKOV‐3, and 65.4 µM at 65 h in A2780 cells (*p* < 0.01). Comparing the dose‐ and time‐dependent antiproliferative effect of EA on ovarian cancer cell lines, the results revealed that EA had the most cytotoxic effect on OVCAR‐3 cells compared to A2780 and SKOV‐3 cells. On the other hand, the IC_50_ value was found to be 159.5 µM at 97 h in EA‐treated healthy ovarian epithelial cells (OSE), which indicates that EA did not show a significant cytotoxic effect on normal cells with respect to ovarian cancer cells (*p* < 0.01). After it was determined that the cytotoxic effect of EA was more pronounced in OVCAR‐3 cells, the further analyses were continued with this cell line.

**Table 1:** The half‐maximal inhibitory concentrations (IC_50_) of evernic acid (EA) on cells.

Cell lines	IC_50_ concentration	Time
OVCAR‐3	10 μM	60 h
SKOV‐3	124 μM	68 h
A2780	65,4 μM	65 h
OSE	159,5 μM	97 h

### 2.2 Colony Formation Assay

**Figure 2:**
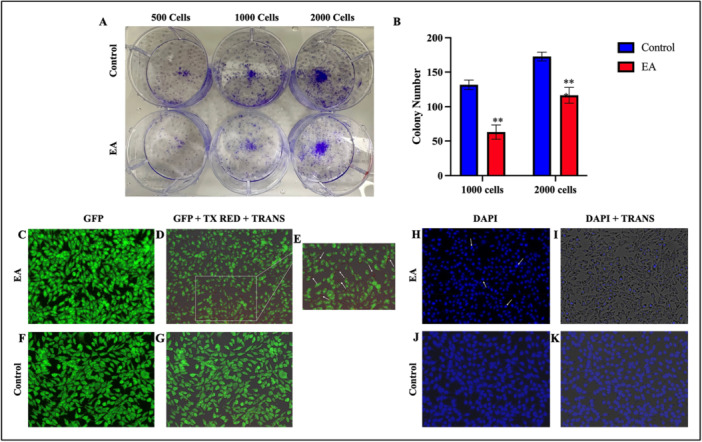
Colony formation assay showing the effect of EA on the colony forming ability of OVCAR‐3 cells. (A) Cells stained with crystal violet and (B) graph of colony formation analysis by number of colonies. Fluorescence microscope image of OVCAR‐3 cells stained with AO (Original microscope magnification = 20x). (C) Image of EA‐treated cells (10 μM) captured with GFP fluorescence channel, (D) Merged image of EA‐treated cells (10 μM) captured with GFP, TX Red and TRANS channels (Z‐Stack), (E) 200% zoomed view of Z‐Stack image, arrows indicate apoptotic OVCAR‐3 cells stained with AO, (F) Image of control cells taken with GFP fluorescence channel, (G) merged image of control cells taken with GFP, TX Red and TRANS channels (Z‐Stack). DAPI staining image of OVCAR‐3 cells. (Original microscope magnification = x20). (H) Image of EA‐treated cells taken with DAPI fluorescence channel, arrows indicate fragmented apoptotic OVCAR‐3 cells showing intense fluorescence, (I) merged image of EA‐treated cells taken with DAPI and TRANS channels (Z‐Stack), (J) image of control cells taken with DAPI fluorescence channel, (K) merged image of control cells taken with DAPI and TRANS channels (Z‐Stack). (**p < 0.01, ***p < 0.001).

The effect of EA on the proliferative capacity of OVCAR‐3 cells was examined using a colony formation assay to assess their sensitivity to EA, as well as their long‐term survival and colony‐forming ability. The assay evaluates a single cell's ability to divide indefinitely by measuring its capacity to produce colonies. Figure 2A,B show the results of the colony formation assay using the fluorescent properties of crystal violet dye. In the assay, 10 µM of EA treatment has been made starting with three different numbers (500, 1000, and 2000) of OVCAR‐3 cells. A statistically significant decrease of colony formation was determined when 1000 (*p* < 0.01) and 2000 (*p* < 0.01) OVCAR‐3 cells were initially inoculated with an IC_50_ concentration of EA. Considering the decreased number of colonies after EA treatment, it was determined that 10 μM EA treatment decreased the colony formation ability of OVCAR‐3 cells.

### 2.3 Acridine Orange (AO) Staining

Acridine Orange (AO) staining is effectively used to determine the differences between living, apoptotic, and necrotic cells. In the AO staining experiment, green fluorescence is observed in the nuclei of live cells due to the binding of acridine orange to DNA, while apoptotic cells are smaller, denser, and round in shape, and orange/red fluorescence is observed in their nuclei [41, 42]. AO staining is effectively used to determine the differences between living, apoptotic, and necrotic cells. During the early phases of apoptosis, the capacity of the lysosomes to store acridine orange is completely unaffected, but during necrosis, it is instantly lost. This distinction can be used to distinguish between necrotic and apoptotic cells. When acridine orange binds to DNA monomerically, it produces green fluorescence; when it binds to lysosomes polymerically, it produces red fluorescence. Because the lysosomal membrane remains intact during apoptosis, the red fluorescence remains unchanged, but the green fluorescence may decrease as a result of DNA degradation, leading to a net increase in the red signal in apoptotic cells. Figure 2C–G shows the fluorescence microscope images of 10 µM EA‐treated and untreated OVCAR‐3 cells stained with AO. Red fluorescence was observed more intensely in the nuclei and cytoplasm of OVCAR‐3 cells treated with 10 μM (IC_50_) EA with respect to the control group. According to the results, there was DNA damage and the initiation of the apoptosis process in EA‐treated OVCAR‐3 cells.

### 2.4 DAPI Staining

DAPI staining was performed to visualize nuclear morphology and cell viability to determine the EA's effect on the OVCAR‐3 cell line. DAPI binds to DNA and causes staining of the cell nucleus; therefore, it is expected that the nucleus in apoptotic cells will be denser and fragmented. DAPI (4,6‐diamidino‐2‐phenylindole) is a fluorescent dye that firmly binds itself to adenine and thymine‐rich DNA regions. DAPI serves as a signal for membrane viability and can be used to stain both live and fixed cells since it can penetrate an intact cell membrane. According to DAPI staining images, sharp fluorescence was observed in the nucleus of untreated OVCAR‐3 cells, indicating live cells (Figure 2H,I). On the other hand, treatment with 10 µM EA resulted in apoptosis in the OVCAR‐3 cells since there was decreased fluorescence uptake compared to the control group. In addition, it was observed that control cells had a uniform structure and an oval shape, while EA‐treated cells had apoptotic nuclear damage (Figure 2J,K).

### 2.5 Wound‐Healing Assay

**Figure 3:**
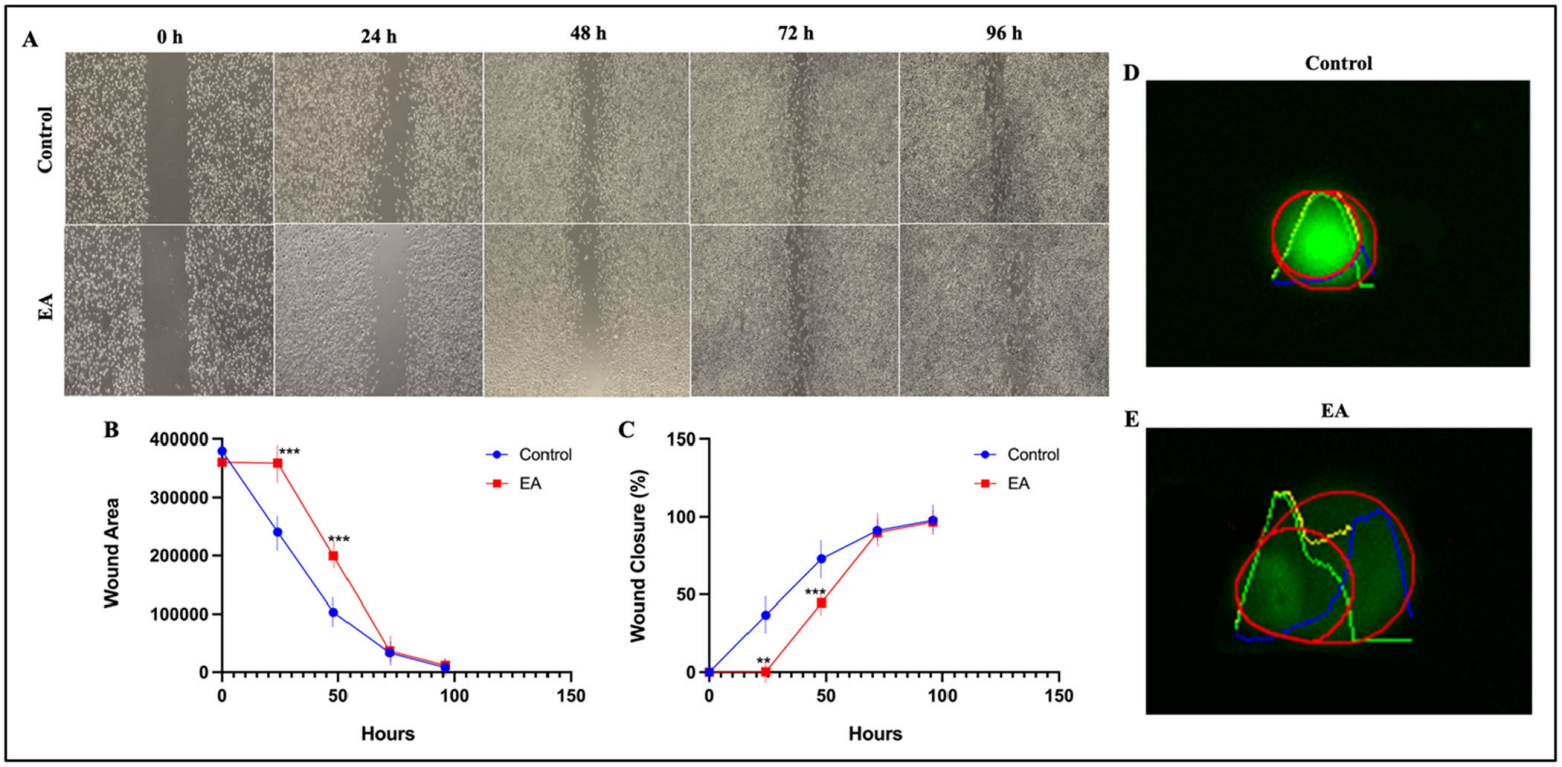
Wound healing assay showing the wound healing effect of EA on OVCAR‐3 cells. (A) Inverted microscope images (Olympos, CKX53) of the wound closure potential of cells with and without EA treatment. (B) Wound healing analysis graph showing the wound healing effect of EA on OVCAR‐3 cells with the reduction of scar area and (C) wound healing analysis graph showing the % wound closure effect. Comet Assay images of OVCAR‐3 cells by OpenComet. (Original microscope magnification = x20). (D) Control group comet image and tail information (E) 10 μM (IC_50_) EA‐treated OVCAR‐3 cell group comet image and tail information. (**p < 0.01, ***p < 0.001).

The wound‐healing assay is a method to study cell migration profiles In Vitro. The potential of EA treatment on OVCAR‐3 cells migration ability was evaluated by wound healing assay. The microscope images of EA‐treated and untreated cells at 0, 24, 48, 72, and 96 h were used in the study. Wound closure rate and time‐dependent decrease in wound areas after EA treatment were presented in Figure 3A–C. A significant decrease in the rate of wound area closure was observed in OVCAR‐3 cells after EA (IC_50_, 10 μM) treatment compared to the control. Specifically, the wound closure rate increased by 36% and 28% after 24 h (*p* < 0.01) and 48 h (*p* < 0.01 and *p* < 0.001), respectively. On the other hand, no statistically significant change was observed in the wound closure rate at 72 and 96 h. These findings indicate that EA causes a decrease in the migration ability of OVCAR‐3 cells within 48 h.

### 2.6 Genotoxicity Analysis

To evaluate the genotoxic effect due to EA application on OVCAR‐3 cells, a comet assay was performed. OVCAR‐3 cells were exposed to 10 µM EA for 60 h. Comet images were illustrated in (Figure 3D,E and Table 2). The images were taken with a fluorescence microscope and were analyzed with the OpenComet plugin in the ImageJ program. In the genotoxicity analysis experiment, DNA damage was observed in 10 μM (IC_50_) EA‐treated OVCAR‐3 cells compared to the untreated control group. As shown in Figure 3D,E and Table 2, head and tail intensities were significantly lower in the 10 µM EA‐treated group (*p* < 0.01). In addition, tail length and movement were significantly increased (*p* < 0.05) due to the EA application, which indicates DNA damage in OVCAR‐3 cells.

**Table 2:** Mean and SD values of measurements for DNA damage of EA‐treated and control group.

Parameters	Control group (*n* = 51)	EA‐treated group (IC_50_) (*n* = 87)
*Head length (μm)*		
Normal	26.98 ± 13.39	29.74 ± 15.94
Normal + Outlier	27.69 ± 13.66	30.75 ± 16.57
*Tail length (μm)*		
Normal	8.55 ± 13.72	13.65 ± 16.41
Normal + Outlier	8.73 ± 13.72	14.30 ± 16.83
*Head intensity (%)*		
Normal	136.50 ± 61.35	80.65 ± 37.58
Normal + Outlier	135.37 ± 61.08	80.09 ± 37.20
*Tail intensity (%)*		
Normal	68.59 ± 35.79	47.76 ± 19.98
Normal + Outlier	69.02 ± 36.61	48.56 ± 20.18
*Tail moment*		
Normal	7.64 ± 13.85	11.70 ± 15.31
Normal + Outlier	7.58 ± 13.63	12.16 ± 15.68
*Olive moment*		
Normal	4.42 ± 7.12	7.46 ± 9.07
Normal + Outlier	4.51 ± 7.10	7.74 ± 9.31

### 2.7 Cell Cycle Analysis

**Figure 4:**
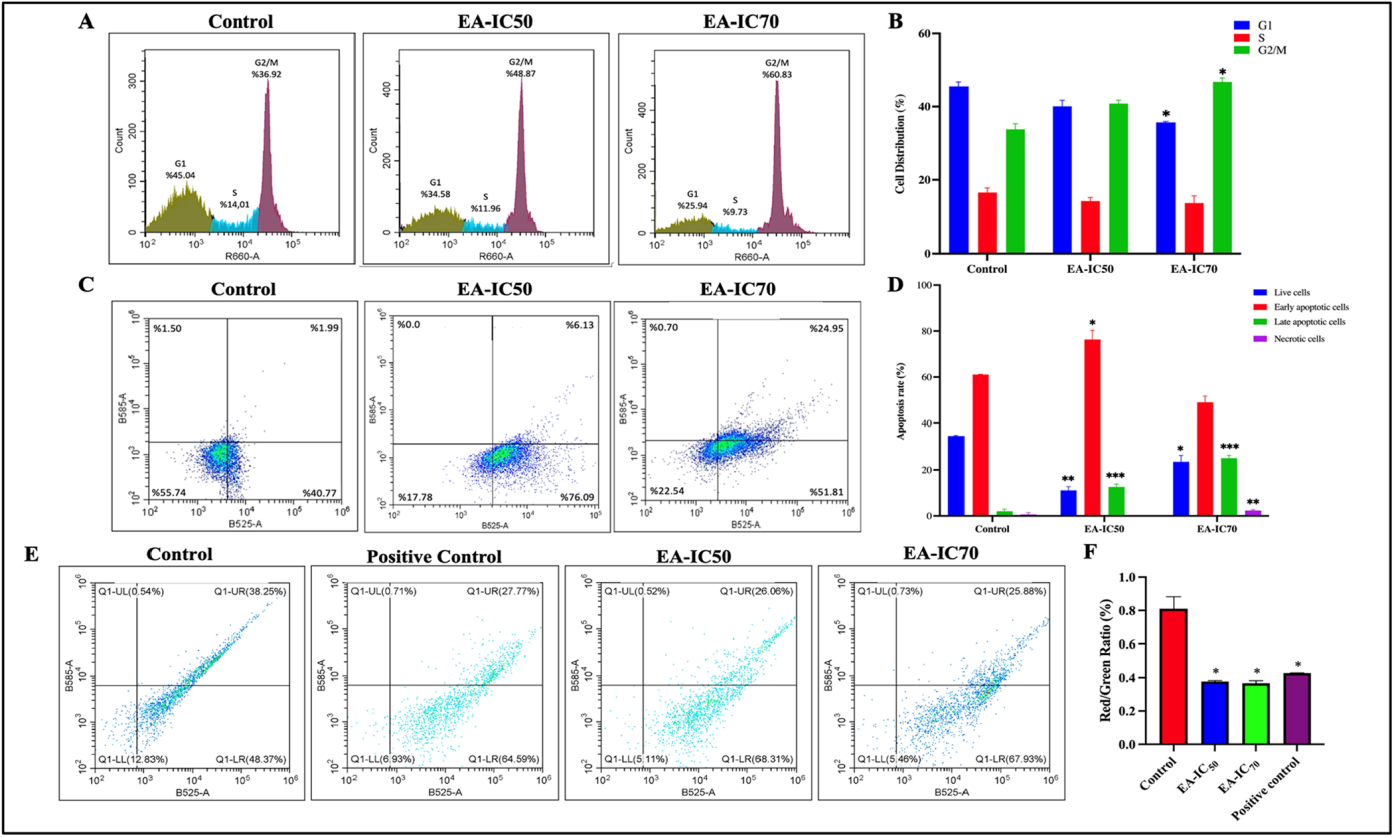
(A) Cell cycle scatter plots obtained by flow cytometry in control, EA‐IC_50_, and EA‐IC_70_‐treated OVCAR‐3 cells. (B) Statistical representation of each phase in response to EA incubation in OVCAR‐3 cells. Data were obtained in triplicate and visualized as the mean ± SD of the experiment. (C) Apoptosis scatter plots obtained by flow cytometry in control, EA‐IC_50_ and EA‐IC_70_‐treated OVCAR‐3 cells. (D) Statistical representation of each phase in response to EA incubation in OVCAR‐3 cells. Data were obtained in triplicate and visualized as the mean ± SD of the experiment. (E) Mitochondrial membrane potential plots obtained by flow cytometry in control, positive control, and EA‐IC_50_ and EA‐IC_70_‐treated OVCAR‐3 cells. (F) Red/green fluorescence ratio proportional to cell viability in response to EA incubation in OVCAR‐3 cells. (*p < 0.05, **p < 0.01, ***p < 0.001).

The effect of EA at IC_50_ (10 µM) and IC_70_ (25 µM) concentrations on the cell cycle distribution over OVCAR‐3 cells was analyzed by flow cytometry. Cell cycle distribution plots of untreated control, EA‐IC_50_ treated, and EA‐IC_70_ treated cells were shown in Figure 4A,B. According to the results, EA causes cell cycle arrest in OVCAR‐3 cells in the G2/M phase by 20.87% in the EA‐IC_50_ treated group and 38.39% in the EA‐IC_70_ treated cells compared to the control group. The results were statistically significant for the EA‐IC_70_ treated group. 25 μM (IC_70_) EA treatment decreased cell accumulation in G1 and S phases in OVCAR‐3 cells, while it significantly increased (*p* < 0.05) cell accumulation in the G2/M phase, indicating cell cycle arrest in that phase. These results indicated that 25 µM EA treatment induces cell cycle arrest in the G2/M phase and inhibits the transition of cells to mitosis, thus playing a role in cell cycle regulation.

### 2.8 Apoptosis Analysis

Annexin V and propidium iodide (PI) assays by using flow cytometry were performed to identify cell death due to EA treatment. In 10 μM (IC_50_) EA‐treated cells, the number of live cells decreased by 17.78%, early apoptotic cells increased by 76.09%, and late apoptotic cells increased by 6.13%. On the other hand, in 25 μM (IC_70_) EA‐treated cells, the number of live cells decreased by 22.54%, early apoptotic cells increased by 51.81%, and late apoptotic cells increased by 24.95% (*p* < 0.01, *p* < 0.001). The results showed that EA triggers cell death by activating apoptotic mechanisms in OVCAR‐3 cells in a concentration‐dependent manner.

### 2.9 Mitochondrial Membrane Potential (MMP/ΔΨm) Assay

Mitochondrial membrane potential (MMP/ΔΨm) is an important parameter of mitochondrial function and is used as an indicator of cell health. In dead cells with high MMP, JC‐1 forms complexes known as JC‐1 aggregates and emits an orange‐red fluorescence upon excitation with the appropriate wavelength, whereas in cells with low MMP (live cells), JC‐1 remains in monomeric form and emits a green fluorescence. The effect of EA on MMP in OVCAR‐3 cells was examined by çıkaralım flow cytometry bu deneyin sonucu makalede yer almadığı için çıkaralım. As a result of the study, it was found that the number of dead cells increased in both 10 and 25 μM EA‐treated cells compared to the control group (*p* < 0.05) (Figure 4E,F). In addition, flow cytometry analysis revealed that the red/green fluorescence ratio, which is directly proportional to cell viability, decreased by 51% in EA‐treated cells at IC_50_ concentration (10 μM) and by 54% in EA‐treated cells at IC_70_ concentration (25 μM) compared to the control group (*p* < 0.05). In addition, the cell viability was decreased 47% compared to the positive control (*p* < 0.05). The results show that EA induces apoptosis by targeting mitochondrial functions that play a critical role in cellular energy metabolism and increases cell death by modulating MMP. The changes induced by EA on MMP in OVCAR‐3 cells lead to impaired mitochondrial function and decreased cellular viability. These effects of EA are thought to increase anticancer activity by inducing cell death through mitochondrial pathways. The data obtained provide important evidence that EA can be used as a potential therapeutic agent through mitochondrial targeting.

### 2.10 Gene Expression Analysis

**Figure 5:**
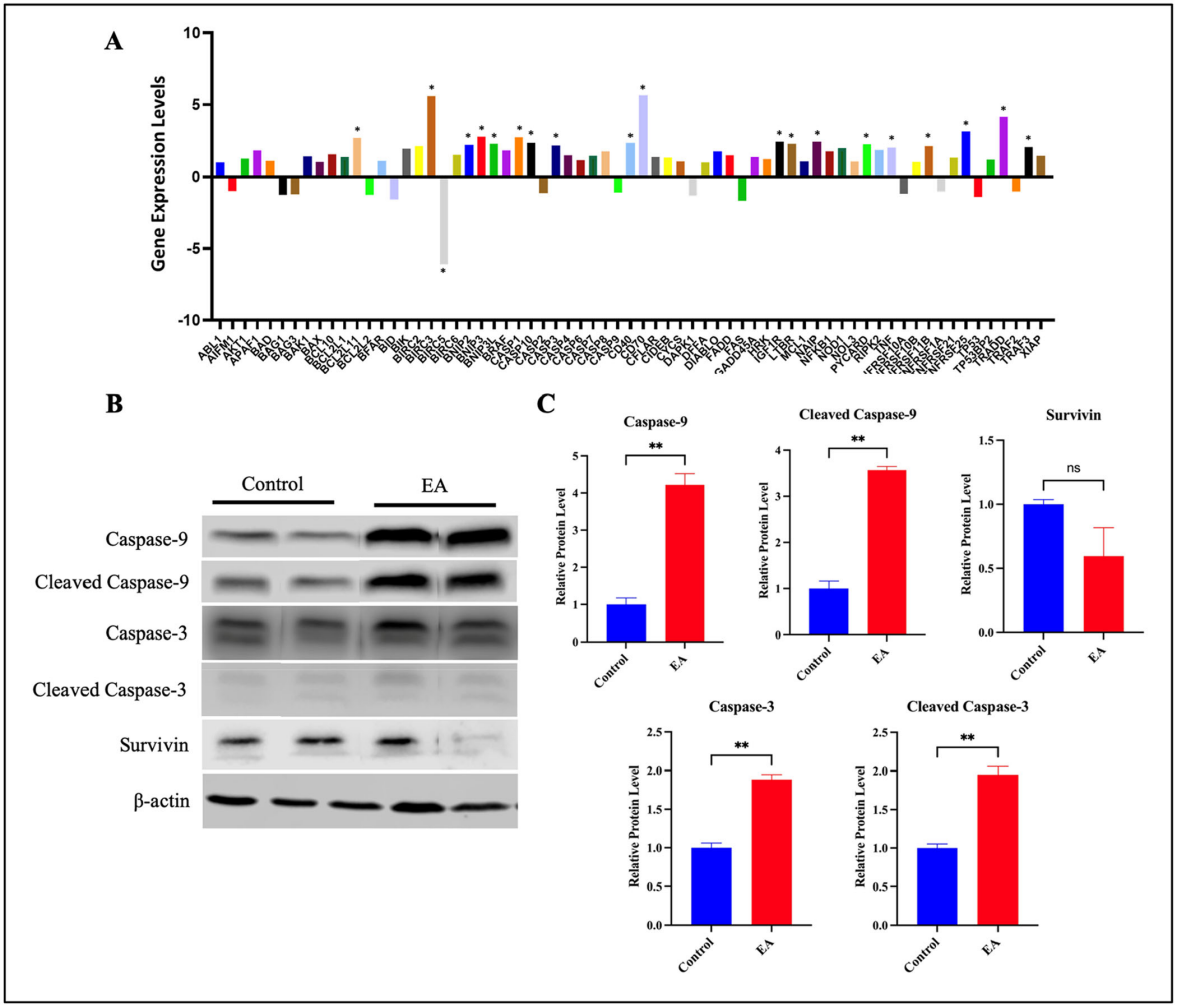
(A) Gene expression levels of apoptosis key genes showing significant expression change in EA‐treated OVCAR‐3 cells. (B) Protein expression levels caspase‐3, caspase‐9 and survivin proteins by western blot analysis, (C) the signal intensity graphs of respective Western blots. (*p < 0.05).

In the gene expression analysis, we selected 88 genes related to apoptosis as shown in Table 3. According to the results, due to the application of 10 µM EA to OVCAR‐3 cells, the expression levels of 21 genes out of 88 had changed significantly (*p* < 0.05) (Figure 5A and Table 4). Among the apoptotic genes that were found to be statistically significant, only the *BIRC5* (−6.11‐fold) gene exhibited a decrease in expression levels (*p* < 0.05), while an increase was observed in the expression levels of the remaining 20 genes.

**Table 3:** Layout for RT_2_ profiler PCR array.

	1	2	3	4	5	6	7	8	9	10	11	12
A	ABL1	AIFM1	AKT1	APAF1	BAD	BAG1	BAG3	BAK1	BAX	BCL10	BCL2	BCL2A1
B	BCL2l1	BCL2L10	BCL2L11	BCL2L2	BFAR	BID	BIK	BIRC2	BIRC3	BIRC5	BIRC6	BNIP2
C	BNIP3	BNIP3L	BRAF	CASP1	CASP10	CASP14	CASP2	CASP3	CASP4	CASP5	CASP6	CASP7
D	CASP8	CASP9	CD27	CD40	CD40LG	CD70	CFLAR	CIDEA	CIDEB	CRADD	CYC5	DAPK1
E	DFFA	DIABLO	FADD	FAS	FASLG	GADD45A	HRK	IGF1R	IL10	LTA	LTBR	MCL1
F	NAIP	NFKB1	NOD1	NOL3	PYCARD	RIPK2	TNF	TNFRSF10A	TNFRSF10B	TNFRSF11B	TNFRSF1A	TNFRSF1B
G	TNFRSF21	TNFRSF25	TNFRSF9	TNFSF10	TNFSF8	TP53	TP53BP2	TP73	TRADD	TRAF2	TRAF3	XIAP
H	ACTB	B2M	GAPDH	HPRT1	RPLP0	HGDC^a^	RTC^b^	RTC	RTC	PPC^c^	PPC	PPC

^a^ Human genomic DNA contamination.

^b^ Reverse transcription control.

^c^ Positive PCR control.

**Table 4:** Genes involved in the apoptotic pathway with statistically significant (*p* < 0.05) changes in expression levels after EA treatment.

Gene	Fold change ± Standard deviation
*CD70*	5.66 ± 0.52
*BIRC3*	5.60 ± 0.13
*TRADD*	4.14 ± 1.78
*TNFRSF25*	3.14 ± 0.78
*BNIP3*	2.75 ± 0.61
*CASP1*	2.70 ± 1.70
*BCL2L11*	2.69 ± 0.27
*IGF1R*	2.42 ± 1.04
*NAIP*	2.41 ± 0.48
*CD40*	2.36 ± 0.35
*CASP10*	2.34 ± 0.47
*BNIP3L*	2.27 ± 0.33
*LTBR*	2.27 ± 0.25
*PYCARD*	2.23 ± 3.86
*BNIP2*	2.20 ± 0.32
*CASP3*	2.17 ± 0.12
*BIRC2*	2.11 ± 0.23
*TNFRSF11B*	2.10 ± 0.41
*TRAF3*	2.04 ± 0.40
*TNF*	2.01 ± 0.23
*BIRC5*	0.16 ± 0.19

Apoptosis is a regulated process of cell death, and it is activated by two main pathways, intrinsic (mitochondrial) and extrinsic. The intrinsic pathway is primarily regulated by the BCL2 family of proteins and is triggered by cellular stress, leading to mitochondrial outer membrane permeability (MOMP) and subsequent activation of caspases [43]. The BCL2 gene family synthesizes both proapoptotic and antiapoptotic proteins. Significant increases were observed in the expression levels of *BNIP2* (2.20‐fold), *BNIP3* (2.75‐fold), and *BNIP3L* (2.27‐fold) (Table 4), members of the BCL2 family involved in the intrinsic pathway and mitochondrial dysfunction (*p* < 0.05).

The extrinsic pathway is initiated by death receptors on the cell surface, leading to caspase activation. Members of the TNF receptor family primarily function in the extrinsic pathway. Interaction of the death domains on adaptor proteins such as TNF receptor‐associated death domain (TRADD) with the corresponding death domains on the TNF receptors (see Supporting Information S1: Figure 1) facilitates the transmission of the death signal and the activation of apoptotic pathways [44]. There were significant increases in TNF receptor family member genes; *CD70* (5.66‐fold), *TRADD* (4.14‐fold) *TNFRSF25* (3.14‐fold), *CD40* (2.36‐fold), *TNFRSF11B* (2.10‐fold) and *TNF* (2.01‐fold) in the EA treated OVCAR‐3 cells (*p* < 0.05).

The caspase gene family plays a role in both silelim intrinsic and extrinsic pathways of apoptosis. The sequential activation of caspases plays a central role in the apoptosis mechanism. As a result of the study, it was determined that there was a significant increase in the gene expression levels of *CASP1* (2.70‐fold), *CASP3* (2.17‐fold) and *CASP10* (2.34‐fold) genes (*p* < 0.05) (Table 4). CASP1 induces cell apoptosis and is important for the inflammatory response [45]. CASP10 activates CASP3, CASP7, and other caspases (CASP4, CASP6, CASP8, CASP9) responsible for the execution of apoptosis. CASP3 is activated by CASP9 in the intrinsic pathway of apoptosis and by CASP8 in the extrinsic pathway and plays an important role in the execution of apoptosis [43, 44, 46].

The inhibitor of apoptosis (IAP) gene family members prevent apoptosis‐induced cell death by encoding antiapoptotic proteins involved in intrinsic and extrinsic apoptosis pathways [47]. By inhibiting the activity of caspases involved in both pathways, they prevent the execution of apoptosis. An increase in the expression of the IAP family members *BIRC2* (2.11‐fold), *BIRC3* (5.60 fold olacak), and *NAIP* (2.41‐fold) (*p* < 0.05) has been observed as a result. BIRC2 and NAIP (also referred to as BIRC1) specifically inhibit the activity of CASP3 and CASP7 [48, 49]. Unlike other IAP gene family members, *BIRC5* (−6.11‐fold) showed downregulated of gene expression (*p* < 0.05). BIRC5, also called survivin, inhibits apoptosis by preventing the activation of CASP9, which is activated in the intrinsic pathway [49, 50]. These findings highlight the potential role of EA in modulating both intrinsic and extrinsic apoptotic pathways in OVCAR‐3 cells through the regulation of key apoptotic regulators.

### 2.11 Protein Expression Analysis

The analysis of apoptosis‐related gene expression following EA treatment revealed several key genes with significantly altered expression levels. Among them, BIRC5 was found to be downregulated after EA treatment while *CASP3* was upregulated. Since CASP9 activates CASP3 in the intrinsic pathway of apoptosis, CASP3, CASP9, and BIRC5 were selected to be further assessed for protein expression analysis. Western blot analysis confirmed the protein expressions of caspase‐3, caspase‐9, and BIRC5. Bands corresponding to the predicted molecular weights were detected in samples treated with EA and untreated controls. Beta‐actin was used as intrinsic control and band intensities were normalized. (Figure 5B). Analysis revealed increased levels of both full‐length and cleaved forms of caspase‐3 and caspase‐9 in the treated group compared to the control, and this change was found to be statistically significant. In EA‐treated cells, bands corresponding to full‐length caspase‐9 (47 kDa) and cleaved caspase‐9 (37 kDa) were observed to have a robust appearance compared to control samples; quantitative densitometric analysis revealed about a four‐fold increase in the cleaved form of caspase‐9 (*p* < 0.01, Figure 5 C). The changes in full‐length caspase‐3 (35 kDa) and cleaved caspase‐3 (19 kDa) bands were less pronounced, quantitative densitometric analysis revealed a two‐fold increase in the cleaved form of caspase‐3 (*p* < 0.01, Figure 5C). Additionally, a slight, yet not statistically significant decrease in BIRC5 levels was observed in the treated group compared to the control group (*p* > 0.05, Figure 5C). Densitometric analysis normalized to β‐actin confirmed the marginal nature of the observed decrease. Altogether, protein expression analysis revealed that EA did exert significant changes in the caspase‐3 and caspase‐9, levels.

## 3 Discussion

Ovarian cancer is a serious public health problem due to high mortality rates and limited treatment options [51, 52]. Therefore, the identification of new drug candidates with high therapeutic efficacy and low side effects is crucial for improving ovarian cancer treatment. Small molecules are widely utilized in cancer treatment due to their ability to bind intracellular and extracellular targets effectively, their bioavailability, and their potential for targeted therapy [20, 21, 53]. EA, a natural secondary metabolite obtained from lichen, has been reported to exhibit multiple biological activities, including antitumor, antimicrobial, antiviral, anti‐inflammatory, antioxidant, and anticancer properties [54, 55, 56]. Previous studies have demonstrated the antiproliferative effects of EA on various cancer cell lines, such as prostate cancer (22RV1), colon cancer (HT‐29, LS174), hepatocellular carcinoma (Hep‐G2), glioblastoma (A‐172, T98G), melanoma (FemX), and lung cancer (A549) [28, 57, 58, 59]. However, there is no study investigating the effects of EA on ovarian cancer cells. In this study, the antiproliferative and anticancer effects of EA, a secondary metabolite obtained from lichen, on ovarian cancer cell lines (A2780, SKOV‐3, OVCAR‐3) were evaluated. The results demonstrated that EA exhibited significant biological effects on these cell lines. A notable finding was that EA demonstrated cytotoxic effects on cancer cells without any significant toxicity in normal ovarian surface epithelial cells (OSE), which supports the potential of EA as a targeted therapeutic agent.

Conventional chemotherapeutic agents usually target all rapidly dividing cells, damaging normal tissues such as immune system cells and the gastrointestinal tract [60]. In contrast, the selective effects of molecules such as EA have the potential to improve both treatment tolerability and patient quality of life [37]. Molecules that selectively target cancer cells and do not harm healthy cells include tyrosine kinase inhibitors, monoclonal antibodies, immune checkpoint inhibitors, and agents derived from natural compounds [61, 62, 63]. However, these agents have their own limitations, such as the high cost and complex production processes of monoclonal antibodies and the low bioavailability of some natural compounds [64, 65]. On the other hand, EA has a unique therapeutic potential on cancer cells because it is a natural metabolite derived from lichens, its production is sustainable, and it can affect strategic mechanisms at the molecular level. In addition, OVCAR‐3 is a serious adenocarcinoma‐derived human ovarian cancer cell line with high malignancy potential [10]. This cell line offers an effective research opportunity in advanced ovarian cancer models due to its tendency to develop resistance to chemotherapeutic agents and its rapid proliferation capacity [10]. The cytotoxic effect of EA on OVCAR‐3 indicates that this molecule may be effective against chemotherapy‐resistant cells with high malignancy potential. This finding suggests that EA can be evaluated not only as a potential therapeutic agent in the treatment of ovarian cancer but also as a promising agent for drug‐resistant cancer types.

Apoptosis, a tightly regulated process essential for maintaining cellular homeostasis, is a promising target for cancer therapy. Kızıl et al. (2014) determined that the rate of early and late apoptotic cells increased significantly in EA‐treated HeLa cells. They stated that EA caused a significant increase in the Bax/Bcl‐2 ratio and thus directed the cells to apoptosis [66]. Similar to this evidence in the literature, in this study, flow cytometry analysis showed that EA has been shown to induce apoptosis in ovarian cancer cells. It was determined that EA at the IC_70_ dose caused approximately 70% of OVCAR‐3 cells to undergo apoptosis and a significant cell cycle arrest through regulation of cell‐cycle‐related genes. The colony formation assay evaluates the clonogenic capacity of cancer cells, providing a measure of their ability to survive and proliferate indefinitely. Although Roser et al. reported that EA did not affect colony formation in colon cancer cell lines (HCT‐116) [36], this study revealed that due to the treatment of EA, the colony formation capacity of OVCAR‐3 cells was reduced by approximately 35%. These findings support the results of cell cycle analyses and show the suppressive effect of EA on cell proliferation. This difference can reflect cell line‐specific differences and highlights EA's ability to suppress clonogenic potential in ovarian cancer cells. Such inhibition can result from EA's ability to arrest the cell cycle and induce apoptosis, which further supports its therapeutic potential in limiting cancer progression.

The proapoptotic effect of EA on OVCAR‐3 cells was also shown with AO and DAPI staining analysis. In this study, AO staining revealed that EA‐treated OVCAR‐3 cells exhibited significant apoptotic changes, as indicated by orange/red fluorescence in the nuclei. This fluorescence suggests lysosomal damage and the initiation of apoptosis, consistent with the hallmark features of programmed cell death, such as chromatin condensation and DNA fragmentation [41, 42]. Similarly, DAPI staining highlighted condensed, fragmented nuclei in EA‐treated cells, further supporting its proapoptotic activity [67, 68]. Together, these findings reveal the role of EA in promoting apoptosis at the cellular level and provide insights into its potential as a therapeutic agent.

Cell migration is a critical process in cancer metastasis, and its inhibition represents a pivotal therapeutic target. Wound healing assays are crucial tools for assessing cell migration potential, which plays a vital role in various physiological and pathological processes, such as tissue repair, cancer metastasis, and inflammation. Wound healing assays revealed that EA reduced the migration capacity of OVCAR‐3 cells by 36% at 24 h and 28% at 48 h compared to the control. In the context of wound healing analysis, the gap closure observed after EA treatment of OVCAR‐3 cells provides valuable insights into its potential effects on cell migration. When compared to control cells, which exhibit a natural rate of migration to close the wound, the EA‐treated cells demonstrate inhibited migration. It could indicate that EA hinders migration, possibly by affecting cellular motility or inhibiting crucial processes such as matrix remodeling or actin filament dynamics. A comparison of the treatment group to control cells also allows for the identification of dose‐dependent effects, providing further understanding of EA's mechanism of action. Overall, analyzing the rate and extent of gap closure in treated versus control cells can reveal important therapeutic implications, especially in the context of conditions where cell migration plays a key role, such as cancer metastasis. These results are consistent with previous studies demonstrating EA's suppression of cell migration in breast cancer cells (MCF‐7, MDA‐MB‐453) [37]. EA contributes to preventing the spread of ovarian cancer by inhibiting cell migration, and these findings make EA a valuable candidate for antimetastatic therapies, as it has the potential to induce apoptosis and suppress cancer metastasis, similar to many anticancer agents used in routine treatment.

The different expression levels of the genes involved in the apoptosis pathway after EA application reveal the complex regulatory mechanisms of apoptosis in OVCAR‐3 cells. Increased expression levels of *CASP3* lead to induction of apoptosis through both intrinsic and extrinsic pathways [69]. CASP9 activates CASP3 in the intrinsic (mitochondrial) pathway of apoptosis, whereas BIRC5 (Survivin) inhibits the activation of both CASP3 and CASP9 [49, 50]. The decrease in the expression level of *BIRC5* indicates that the capacity of cells to inhibit apoptosis is reduced, and the sensitivity of EA‐treated OVCAR‐3 cells to apoptotic stimuli may potentially increase. The increased expression of *CASP1*, *CASP3*, and *CASP10* genes indicates activation of both intrinsic and extrinsic apoptosis pathways. Conversely, the observed downregulation of BIRC5 suggests reduced antiapoptotic capacity in EA‐treated cells, enhancing their sensitivity to apoptotic stimuli [49, 50, 69]. These results underline EA's potential to selectively induce apoptosis in cancer cells, offering a dual approach to cancer treatment.

Further analysis of mitochondrial dysfunction, a critical factor in apoptosis regulation, revealed approximately 50% reduction in MMP in EA‐treated cells compared to the control. This mitochondrial dysfunction, combined with increased CASP3 and CASP9 cleavage, highlights EA's role in activating the intrinsic apoptosis pathway. The robust appearance of cleaved CASP9 bands implies that EA promotes mitochondrial membrane permeabilization and subsequent apoptosome formation. The trends observed in the western blot data provide valuable insights. Specifically, the activation of cleaved CASP3 and CASP9 suggests that EA treatment may modulate apoptotic pathways in OVCAR‐3 cells. Survivin, (IAP) family, is commonly overexpressed in cancer cells and inhibits apoptosis by directly targeting key effectors such as CASP3 and CASP9 [70]. In the western blot analysis, 1.8‐fold decrease was observed for survivin protein levels. This indicates that apoptosis can occur without significant changes in survivin protein expression, highlighting the complexities of apoptotic regulation. In the western blot analysis, approximately two‐fold and four‐fold increases were observed for CASP3 and CASP9 protein levels, respectively. These results suggest that EA can exert its effects at several levels of gene regulation, including mRNA stability, protein translation, or protein degradation. Even if the level of gene expression is suppressed, changes in the regulatory mechanism may lead to increased protein expression levels. In conclusion, the positive effect of EA on proapoptotic proteins such as CASP3 and CASP9 may trigger the death of cancer cells.

The cellular‐scale effects of EA, such as the inhibition of colony formation, the decrease in cell migration, the increase in apoptotic cell rates, the induction of cell cycle arrest in the G2/M phase, and the decrease in MMP, suggest that EA may play an important role in the suppression of the proliferation and invasiveness of ovarian cancer cells. Cell cycle analyses support the hypothesis that EA inhibits the proliferation of cancer cells by targeting mitotic checkpoints. Furthermore, analysis of the molecular mechanisms underlying apoptosis revealed that EA triggered significant changes in the expression of apoptosis‐related genes and proteins, specifically suppressing the antiapoptotic *BIRC5* gene and activating the proapoptotic *CASP3* gene, thereby strongly supporting the hypothesis that EA triggers apoptotic cell death mechanisms.

A significant finding of this study is that it also revealed the genotoxic effects of EA. An increase in genetic instability in cancer cells is an important mechanism that may contribute to an increase in sensitivity to treatment. The fact that EA induces apoptosis by causing DNA damage in cancer cells suggests that it may create synergistic effects in combination treatments with potential chemotherapeutic agents. In addition, the low toxicity profile of EA compared to chemotherapy drugs offers an important advantage, especially in terms of reducing chemotherapy‐related adverse effects. The integration of these findings with EA's proapoptotic effects provides a comprehensive view of its mechanism of action, further supporting its therapeutic applicability. Kalın et al. revealed the antiproliferative effects of EA on human breast cancer cells (MCF‐7) and reported that this effect triggered apoptotic mechanisms through DNA damage [37]. In another study, ellagic acid, a bioactive polyphenolic compound found naturally as a secondary metabolite in many plant taxa, was reported to induce DNA damage and promote apoptosis in various cancer cell lines while exhibiting minimal toxicity in healthy cells. It has also been reported that ellagic acid exhibits anticancer effects by targeting the regulation of inflammatory pathways such as NF‐κB and COX‐2 along with genotoxic effects [71, 72, 73].

The results of our study showed that EA exerts a marked proapoptotic effect on ovarian cancer cells. This was evidenced by significant changes in the expression levels of key apoptosis‐related genes and proteins, in particular the upregulation of *CASP3* and the downregulation of *BIRC5*. *CASP3* is an important executioner caspase in the apoptotic cascade, while *BIRC5* is known to inhibit apoptosis by promoting cell survival. The observed increase in *CASP3* expression together with the suppression of *BIRC5* strongly suggests a shift toward apoptotic signaling in response to EA treatment. Moreover, the reduction in mitochondrial membrane potential further supports the involvement of the intrinsic (mitochondrial) apoptotic pathway. Disruption of mitochondrial integrity typically leads to the release of cytochrome c and subsequent activation of the caspase cascade. Taken together with the G2/M phase cell cycle arrest observed in the study, these findings suggest that EA not only stops uncontrolled proliferation but also actively induces programmed cell death. In addition, the genotoxic effects detected by the comet assay suggest that DNA damage may be a contributing factor in the initiation of apoptosis. DNA integrity is critical for cell survival and damage beyond the repair capacity of the cell may trigger apoptotic mechanisms. In conclusion, EA was found to activate apoptotic signaling pathways at both the genetic and functional levels and to effectively induce apoptosis in ovarian cancer cells. Importantly, the absence of cytotoxic effects on normal ovarian epithelial cells highlights the selective toxicity and potential therapeutic value of EA as a candidate agent for the treatment of ovarian cancer.

This study reveals the therapeutic potential of EA in ovarian cancer treatment due to its significant antiproliferative and proapoptotic effects on ovarian cancer cells, particularly the OVCAR‐3 cell line. The ability of EA to induce apoptosis through genotoxic effects, regulate mitochondrial membrane potential, and inhibit cell migration suggests that it has a multifaceted mechanism of action. The current literature on the anticancer effects of EA is limited, and the findings of this study demonstrate for the first time that EA has potent therapeutic effects on ovarian cancer cells. These findings suggest that EA is a promising candidate for targeted cancer therapies and has the potential to improve treatment efficacy and patient quality of life while minimizing side effects. Unlike chemotherapeutics used in the routine treatment of ovarian cancer patients, the potential of the EA molecule, which is found in nature and can be easily synthesized in the laboratory, as a drug candidate molecule has been revealed In Vitro.

## 4 Conclusion

The findings of the present study demonstrated that EA exhibited an antiproliferative effect on ovarian cancer cells, which was mediated by the apoptosis pathway at both the cellular and molecular levels. To transfer the current findings to clinical applications, the efficacy and safety profile of EA should be evaluated more comprehensively in In Vivo models and further preclinical studies. Furthermore, further elucidation of the molecular mechanisms of EA provides an important opportunity to develop targeted cancer therapy strategies. In conclusion, the use of EA alone or in combination with chemotherapeutic agents may be considered a promising alternative in ovarian cancer treatment.

## 5 Experimental

### 5.1 Cell Culture

The human ovarian carcinoma cell lines, A2780, SKOV‐3, OVCAR‐3, and the human ovarian surface epithelial cells (OSE), were cultured in a 5% CO_2_‐containing incubator at 37°C. Each cell line was cultured in its specific complete medium (OVCAR‐3: Dulbecco's Modified Eagle Medium (DMEM) (Gibco, Thermo Fisher Scientific Inc.), supplemented with 20% (v/v) FBS (Gibco, Thermo Fisher Scientific Inc.), 1% penicillin‐streptomycin and 10% insulin, A2780: RPMI‐1640 Medium (Gibco, Thermo Fisher Scientific Inc.), supplemented with 10% (v/v) FBS, 1% penicillin‐streptomycin and 1% l‐glutamin, SKOV‐3: DMEM, supplemented with 10% (v/v) FBS and 1% penicillin‐streptomycin, OSE: McCoy's 5 A Medium (Gibco, Thermo Fisher Scientific Inc.), supplemented with 10% (v/v) FBS, and 1% penicillin‐streptomycin).

### 5.2 Preparation of Evernic Acid

EA was purchased from Santa Cruz Biotechnology (Cat. No. Sc‐294581). 100 μM EA was prepared in DMEM medium at a rate not exceeding 0.05% DMSO. Then, EA stock solution was diluted with appropriate media, and different concentrations were prepared as 50, 25, 12.5, 6.25, 3.125, and 1.56 μM.

### 5.3 xCELLigence Real‐Time Cell Analysis (RTCA)

The antiproliferative effect of EA on A2780, SKOV‐3, OVCAR‐3, and OSE cells was analyzed using the xCELLigence RTCA DP system (ACEA Biosciences, San Diego, CA, USA). After background measurement, A2780 cells were seeded at 2 × 10^4^ cells/100 μL, and SKOV‐3, OVCAR‐3, and OSE cells were seeded at 1 × 10^4^ cells/100 μL in a 16‐well e‐plate. The plate was placed in the instrument and incubated in a CO_2_ incubator for a period of 24 h. A volume of 100 μL of EA was then added to the wells of the E‐plate at various concentrations (1.56, 3.125, 6.25, 12.5, 25, 50, and 100 μM). Readings were taken every 15 min while the instrument operated for 120 h. On the program, the proliferation curve was monitored in real time. IC_50_ concentrations of EA were measured with the xCELLigence RTCA Software Lite (2.0) program.

This allowed for a comparative analysis of cell growth and viability between the control and EA‐treated ovarian cancer and normal cells by measuring the cellular index intermittently over time. After determining that the antiproliferative activity of EA was highest on OVCAR‐3 cells, further analyses were continued with this cell line.

### 5.4 Colony Formation Assay

OVCAR‐3 cells were seeded in 6‐well plates at a density of 500 cells/well, 1000 cells/well, and 2000 cells/well and cultured in a 5% CO_2_, incubator at 37°C for 7 days to promote colony formation. After 24 h, 10 μM (IC_50_) of EA treatment was applied to the cells and were incubated for an additional 60 h. The plate was gently washed with cold PBS, and the colonies were fixed with methanol. Then, the colonies were stained with 0.5% crystal violet. Following the removal of excess dye, colony formation was quantified by using ImageJ software.

### 5.5 Acridine Orange (AO) and DAPI Staining

OVCAR‐3 cells were cultured in two six‐well plates, which one for AO and the other for DAPI staining, at 5×10^5^ cells per well and incubated for 24 h. After 24 h, 10 μM (IC_50_) of EA treatment was applied to the cells, and they were incubated for an additional 60 h. After that, the medium was aspirated, and the wells were washed twice with PBS. To fix the cells, 1 mL of cold methanol was added, and the cells were incubated at −20°C for 2 min. The cells were washed three times with PBS. Acridine Orange (1 mg/mL) dye was applied and incubated in the dark for 30 min, followed by a final wash with PBS. DAPI (1 mg/mL) solution was applied to the additional six‐well plate and incubated in the dark for 2 min. Imaging of the stained cells was performed using a fluorescence microscope (EVOS M5000 Imaging System, Thermo Fisher Scientific, MA, USA).

### 5.6 Wound‐Healing Assay

OVCAR‐3 cells were seeded in six‐well plates at a density of 2.5 × 10^5^ cells/well, incubated overnight in a CO_2_ incubator at 37°C to form a confluent monolayer, and scratches were made on the cell monolayer using a 200 μL pipette tip. Then scratched OVCAR‐3 cells were treated with 10 μM (IC_50_) of EA, and the cell migration rate was monitored and recorded at 24 h intervals up to 96 h by Olympus CKX53 (CKX53, Olympus, Tokyo, Japan) inverted light microscope. The quantification of the gap size with respect to time was performed using the ImageJ program.

### 5.7 Genotoxicity Analysis

OVCAR‐3 cells were cultured in a six‐well plate at 5 × 10^4^ cells per well and incubated for 24 h. After 24 h, 10 μM (IC_50_) of the EA was applied to the cells, and they were incubated for an additional 60 h. For the comet assay, 50 µL of cell suspension was used for each well, and the cells were incubated in lysis buffer according to the kit protocol (OxiSelect 96‐Well Comet Assay Kit). After lysis, the wells were incubated with alkaline solution. Electrophoresis buffer was prepared and pre‐cooled at 4°C. The wells were placed in the electrophoresis device, and electrophoresis was performed at the voltage and time according to the kit instructions. In the staining step, the wells were washed with PBS after electrophoresis and stained with DNA dye. DNA damage in each well was observed and visualized using a fluorescence microscope (EVOS M5000 Imaging System Thermo Fisher Scientific, MA, USA). The determination of the head and tail lengths, % of head and tail intensity, and tail and olive moments, which all indicated the amount of DNA damage, was assessed using image analysis software (ImageJ plugin: OpenComet).

### 5.8 Cell Cycle Analysis

OVCAR‐3 cells (5 × 10^5^ cells/well) were seeded into a six‐well plate, incubated for 24 h, and treated with 10 μM (IC_50_) and 25 μM (IC_70_) EA. After the EA incubation period, the wells were washed with PBS, and cells were harvested by using 300 µL of trypsin. The cells were centrifuged for 5 min at 1800 rpm Then, 5 mL 70% ethanol was applied to the cells for fixation. After the washing steps, the cells were resuspended in cold PBS, stained with PI (0.5 mg/mL) solution, and incubated in the dark for 15 min. The samples were analyzed by a flow cytometry analyzer (CytoFlex Flow Cytometer, Beckman Coulter Inc. (Brea, CA, USA) PN B49006AE).

### 5.9 Apoptosis Analysis

The assessment of OVCAR‐3 cell apoptosis was performed using the FITC Annexin V/PI Staining Kit (Biolegend, Cat. No. 640914) according to the manufacturer's protocol. Briefly, 10 μM (IC_50_) and 25 μM (IC_70_) concentrations of EA were applied to the cells (5 × 10^5^ cells/well). After the incubation, the cells were harvested and resuspended with Annexin V Binding Buffer, and 5 μL FITC Annexin V and 10 μL of Propidium Iodide (PI) from the kit were added. The samples were incubated for 15 min and then analyzed with a flow cytometry analyzer (CytoFlex Flow Cytometer, Beckman Coulter Inc. (Brea, CA, USA) PN B49006AE).

### 5.10 Mitochondrial Membrane Potential (MMP/ΔΨm) Assay

Early apoptotic cells were assessed using an MMP assay kit containing JC‐1 (MedChemExpress cat no: HY‐K0601‐100T), a fluorescent probe for rapid and sensitive detection of changes in mitochondrial membrane potential (∆Ψm) of cells. Briefly, OVCAR‐3 cells were seeded in a six‐well plate (2 × 10^5^/well) and treated with 10 μM (IC_50_) EA for 24 h. After incubation, JC‐1 (200 μM) dye was diluted to a final concentration of 2 μM in PBS and incubated in a 37°C, 5% CO_2_ incubator for 15–20 min. The dye was then removed, and cells were scanned by fluorescence microscopy (EVOS M5000 Imaging System Thermo Fisher Scientific, MA, USA). The cells were then suspended in PBS and analyzed by flow cytometry (CytoFLEX Flow Cytometer, Beckman Coulter Inc. USA).

### 5.11 Gene Expression Analysis

OVCAR‐3 cells were treated with 10 μM EA for 24 h, and total RNA was extracted using TRIzol reagent (Invitrogen, 15596026) from both treated and untreated cells. Then, cDNA synthesis was carried out using the iScript cDNA Synthesis kit (Bio‐Rad Laboratories, 1708891). Following cDNA synthesis, a qRT‐PCR experiment was conducted using the RT^2^ Profiler PCR array (QIAGEN, 330231), which includes the Human Apoptosis Primer Library panel containing 96 different primers, 88 of which are apoptosis‐related genes and 8 are housekeeping genes (Table 3). A qRT‐PCR reaction was conducted by using the LightCycler 480 Real Time PCR device (Roche Diagnostics, LightCycler 480, Switzerland). The reaction was initiated by an initial denaturation step at 95°C for 10 min, followed by 45 cycles of 95°C for 15 s and 60°C for 1 min. Each reaction was performed in duplicates. The fold change for apoptotic genes was computed using the $2^{‐\Delta \Delta C_{t}}$ method and evaluated at QIAGEN's GeneGlobe Data Analysis Center.

### 5.12 Protein Expression Analysis

After OVCAR‐3 cells were subjected to treatment with 10 µM EA for 24 h, cells were collected and lysed using the Complete Lysis‐M Reagent Kit (Roche, Cat#04719956001) following the manufacturer's protocol. Protein concentrations were determined using the Bradford assay (Thermo Fisher Scientific, Cat# 23238). Following quantification, protein samples were heated to 95°C for 5 min. Equal amounts of protein (15 µg per sample) were separated on a 12% SDS‐PAGE gel and transferred onto PVDF membranes (Millipore, Cat# IPVH00010) using a semi‐dry transfer system (Bio‐Rad Trans‐Blot Turbo, Cat# 1704150). Membranes were blocked with 5% nonfat milk in TBS‐T (Tris‐buffered saline with 0.1% Tween‐20) for 1 h at room temperature and incubated overnight at 4°C with primary antibodies: Caspase 3 (1:1,000, CST, Cat#14220), Cleaved Caspase‐3 (1:1,000, CST, Cat#9664), Caspase 9 (1:1,000, CST, Cat# 9508), Cleaved Caspase‐9 (1:1,000, CST, Cat#52873), Survivin (BIRC5) (1:1000, Santa Cruz, Cat#sc‐17779), and β‐Actin (1:1,000, St. John's, Cat# STJ91464). Blots were washed and incubated with HRP‐conjugated secondary antibodies (1:10,000, CST, Cat# 7074S and Cat# 7076S) for 1 h. Signal detection was performed using chemiluminescence detection reagent (Advansta, Cat# R‐03027 and Cat# R‐03025) and imaged on Li‐Cor Imaging system (Li‐Cor Odyssey Fc). Band intensities were quantified using the Li‐Cor Image Studio software.

### 5.13 Statistical Analysis

All data obtained were expressed as ± standard error of the mean (SEM). Groups were compared by unpaired two‐tailed Student's *t* test and/or one‐way analysis of variance (ANOVA). *p* ≤ 0.05 was considered statistically significant (*p < 0.05, **p < 0.01, and ***p < 0.001, ****p < 0.0001). All results were visualized with GraphPad Prism 9.0 software.

## Conflicts of Interest

The authors declare no conflicts of interest.

## Supporting information

Supplement file 30.

## Data Availability

The data that support the findings of this study are available on request from the corresponding author. The data are not publicly available due to privacy or ethical restrictions.
