# Supplementary material for: Evernic Acid: A Low‐Toxic and Selective Alternative to Chemotherapeutic Agents in the Treatment of Ovarian Cancer
Source: Arch Pharm (Weinheim). 2025 May 22;358(5):e70015. doi: 10.1002/ardp.70015 (PMC12099196; doi:10.1002/ardp.70015)
Supplement: Supplementary file 1 — Supplement file 30. [file ARDP-358-e70015-s001.docx]

**
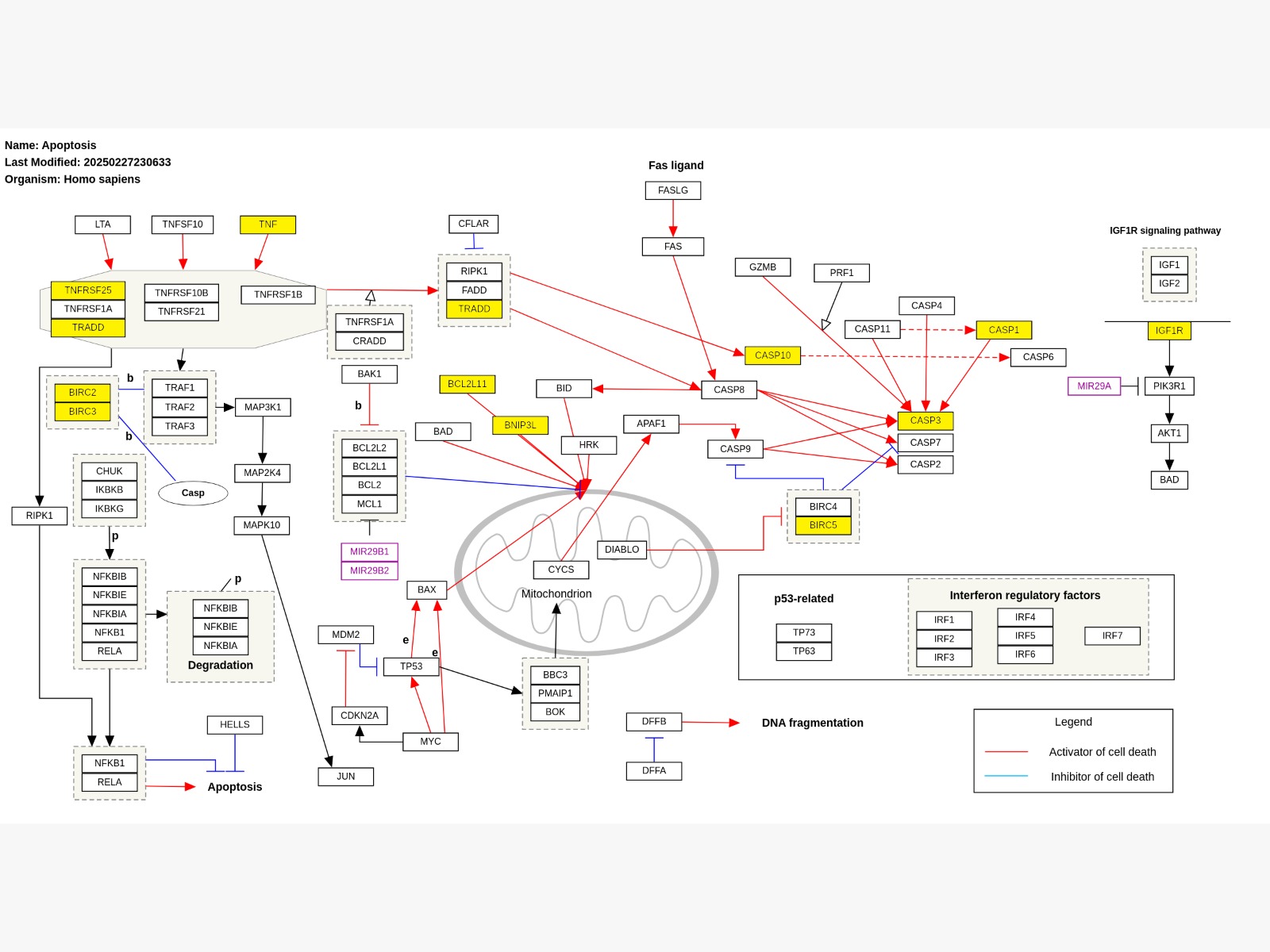
**

**Figure X.** The roles and interaction profiles of genes with statistically significant (*p*<0.05) changes in expression levels in the apoptosis pathway.
